# Polygenic risk scores in the clinic: new perspectives needed on familiar ethical issues

**DOI:** 10.1186/s13073-021-00829-7

**Published:** 2021-01-28

**Authors:** Anna C. F. Lewis, Robert C. Green

**Affiliations:** 1 E J Safra Center for Ethics, Harvard University, 124 Mount Auburn, Street, Cambridge, 02138 USA; 2 Brigham and Women’s Hospital, 75 Francis St, Boston, MA 02115 USA; 3 Ariadne Labs, 401 Park Dr 3rd Floor, Boston, MA 02215 USA; 4 Broad Institute of Harvard and MIT, 415 Main St, Cambridge, MA 02142 USA; 5 Harvard Medical School, 25 Shattuck St, Boston, MA 02115 USA

## Abstract

Clinical use of polygenic risk scores (PRS) will look very different to the more familiar monogenic testing. Here we argue that despite these differences, most of the ethical, legal, and social issues (ELSI) raised in the monogenic setting, such as the relevance of results to family members, the approach to secondary and incidental findings, and the role of expert mediators, continue to be relevant in the polygenic context, albeit in modified form. In addition, PRS will reanimate other old debates. Their use has been proposed both in the practice of clinical medicine and of public health, two contexts with differing norms. In each of these domains, it is unclear what endpoints clinical use of PRS should aim to maximize and under what constraints. Reducing health disparities is a key value for public health, but clinical use of PRS could exacerbate race-based health disparities owing to differences in predictive power across ancestry groups. Finally, PRS will force a reckoning with pre-existing questions concerning biomarkers, namely the relevance of self-reported race, ethnicity and ancestry, and the relationship of risk factors to disease diagnoses. In this Opinion, we argue that despite the parallels to the monogenic setting, new work is urgently needed to gather data, consider normative implications, and develop best practices around this emerging branch of genomics.

## Background

Polygenic risk scores (PRS) are numerical indicators of risk based on multiple genetic markers associated with a disease or trait. Research in this field has recently accelerated, and scores are available for a wide array of traits and conditions, including for conditions such as coronary artery disease, type 2 diabetes, and common cancers [1–5]. Some polygenic reports are already available, both through traditional molecular testing laboratories as ordered by a physician, and through consumer-facing companies [6–8].

In this Opinion, we provide an overview of the state of the science underlying PRS and evidence relevant to their clinical use. We then consider some of the ethical, legal, and social implications (ELSI) familiar to the monogenic setting and genomics scholarship, to ask whether such issues continue to be relevant in the polygenic context. We identify additional concerns and unique challenges by first considering the use of PRS in a public health context and second by discussing long-standing issues with the use of biomarkers that PRS highlight.

## Evidence of PRS potential in disease

Knowledge of the genetics linked to common disease largely comes from comparing cases of a condition to suitably matched controls and looking for variants that are disproportionately present in either group. The variants identified by these genome wide association studies (GWAS) can be combined, in the simplest form in proportion to their effect size, to provide a PRS. Unlike the more expensive sequencing data used in monogenic studies, most of the data available in the polygenic context to date has been from SNP-chips, which cheaply probe a few hundred thousand, sometimes more than a million, of the more common variants within the genome.

For most traits, currently available PRS fall far short of capturing the full variance of a trait as expected from heritability estimates. As larger data sets are studied, variants with smaller effect sizes can be identified, and the scores can capture more of the variance of the trait. New statistical methodologies are also increasing the predictive power of these scores. A review of the construction of PRS is given in Martin et al., where the authors note the ease of producing PRS once the underlying data exists [9].

The scores themselves are normalized within a specific population and hence only give information about where an individual falls within that population, for example within the top 5%. To estimate relative and absolute risks, further work is needed. In addition to giving risk information for developing a disease, some evidence suggests that PRS can predict how likely someone is to respond to a treatment [10–12], for example antidepressants [12, 13].

The underlying GWAS data lends itself to two interlinked problems in moving PRS into the clinic. First, PRS suffer from a *portability problem*. A score based on individuals of one genetic ancestry can be multiple times less predictive in other ancestries [14]—the score does not “port” well across populations. Some of this is because of different patterns of linkage disequilibrium and different allele frequencies in different populations. Most existing data are from individuals of European genetic ancestry, so current scores are most predictive within this population. Note that whereas race and ethnicity are social constructs (either self-reported or assumed on the basis of appearance, for example, by a healthcare provider), genetic ancestry is primarily a statistical concept based on patterns of inheritance, though it nonetheless has ambiguities of interpretation [15]. Compounding this portability issue, the scores are not always accurate even within individuals of the *same* genetic ancestry, but different demographics [16]. How the scores can be applied across groups with different environmental exposures is not well-characterized.

Second, PRS suffer from an *interpretation problem*: we are not yet sure what conclusions can be drawn from them. While some of the predictive signal captured in PRS comes from direct genetic effects, i.e., from variants that influence the phenotype of the individual, other predictive signals are also captured in a standard non-family-based GWAS. These include indirect genetic effects, for example genetic nurture [17]. Effects of assortative mating and environmental confounding are also captured. In addition to these forms of gene-environment correlation, gene-by-environment interactions, where the same variant has a different overall effect in different environments, may also play a role. It is therefore not appropriate to straightforwardly draw causal conclusions from PRS [18].

It is against this context of issues with the basic science of PRS that their potential clinical application needs to be assessed.

## Are PRS ready for the clinic?

In screening and in preventative medicine, the utility of specific tests and models is a complex arena [19]. Some argue that a score has to give very high relative risks to be useful as a screening measure, and PRS are nowhere near this bar [20]. However, others have posited that PRS may already be clinically useful, and at the very least, we should be testing their use in the clinic [1, 21, 22]. Some large scale studies are already underway, for example the WISDOM trial is using PRS and other risk factors to stratify 100,000 women into higher and lower risk for breast cancer [23], and a 20,000 person trial through the eMERGE Network will return a number of PRS for a variety of common conditions to each individual, and track the impact of this result disclosure [24]. The UK government is exploring a plan to return PRS to 5 million individuals through its Accelerating Detection of Disease Initiative [25].

The potential benefits of identifying those at high risk of developing a disease offer multiple approaches to preventative medicine [12]. Primary prevention, avoiding a disease, could be achieved through lifestyle change or proactive treatment. Secondary prevention, detecting a disease early and preventing it from getting worse, could be achieved by more frequent screening of those at high risk (e.g., mammograms for women at high risk of breast cancer) or simply by helping increase uptake and compliance with existing recommended screening [26]. Tertiary prevention, trying to improve quality of life or reducing the symptoms of an existing disease, could be achieved by better knowledge of treatment response, including responses to prescribed drugs. Somewhat more controversially, there could be benefits in identifying those at lower risk, for example in screening these individuals less often and hence saving resources and decreasing risks of over treatment [27]. With the introduction of any novel method to identify those at high risk, the dangers of overdiagnosis (the diagnosis of a condition that would never have caused symptoms or problems) and overtreatment (too much treatment, in particular for insignificant disease) must be considered [28, 29].

Some data already exist for whether these benefits are realizable, but a clear picture is yet to emerge. Two retrospective analyses that integrated coronary disease PRS into absolute risk models found no [30] and a modest [31] statistically significant improvement in accuracy compared to use of the same models without the score. Another study found that when incorporated into risk models, highly elevated PRS were much more predictive of myocardial infarction early in life when other risk factors have yet to manifest [32]. In an early randomized controlled trial of a PRS for coronary heart disease, participants randomized to receive the PRS had improved lipid levels as a result of statin use in those with higher scores [33]. One meta-analysis found little evidence that genetic risk information changed behaviors [34] while others have found evidence for positive behavior change [35, 36]. Finally, a recent study of 7000 individuals in Finland found those with high PRS were more likely to take action such as losing weight or stopping smoking [37].

PRS that are commercially available include those from Myriad Genetics for breast cancer risk [7], from Ambry Genetics for breast cancer and prostate cancer risk [6], and from 23andMe for type 2 diabetes risk [8]. Several companies will produce polygenic reports based on a user’s upload of their 23andMe or Ancestry.com data. Given that there are already PRS on the market, and that large-scale studies investigating the return of PRS have started, reflection on ELSI concerns is urgently needed.

## Are ELSI themes from the monogenic setting relevant?

Genetic information, and its use in the clinic and beyond, has long been considered worthy of special reflection. When the widespread reporting of monogenic variation based on DNA sequencing was on the horizon, a vast amount of work started under the umbrella of ELSI of human genome research. We consider several of the most prominent themes in this literature, to assess their relevance in the polygenic context.

### The relevance of findings to family members

Genetic variation is shared in families, with many ensuing ethical quandaries, running from how to handle surprises about paternity to what to do if you realize that the mother of a baby is a BRCA carrier [38]. An individual has a 50% chance to pass a monogenic variant on to a child, and this binary inheritance can be traced through a family tree. The American Medical Association’s Council on Ethical and Judicial Affairs considered the responsibility of the physician towards a patient’s family members and concluded that the physician has a duty to discuss the significance of genetic information with family members as part of informed consent for genetic testing, including defining the circumstances under which the patient would need to inform family members [39].

It is not clear how these circumstances should be defined for PRS. The PRS of first degree family members are correlated [40], but this information is not as clear cut as in the monogenic case when a precise probability of carrying a variant can be given. How high would polygenic risk need to be to prompt warning to family members? Guidelines developed by professional societies are needed for both patients and providers. These would possibly be condition specific and would need to incorporate input from relevant stakeholders including patients, genetic counselors, epidemiologists, and primary care physicians. Products to serve the separate use case of reproductive planning—the analog of carrier screening for PRS—will likely also surface. Again, guidelines will be needed.

### Defining how to approach secondary or incidental findings

In the monogenic, diagnostic setting of exome and genome sequencing, the American College of Medical Genetics and Genomics (ACMG) has recommended that laboratories look for a defined set of genetic variation that, if present, would be medically actionable [41]. Although this was initially considered controversial [42], it has been adopted in some form by almost all diagnostic sequencing laboratories in the USA. Most PRS are currently generated from SNP-chips that are not suited to capture all of this variation, but it is possible to select SNP-chips that contain much of this variation (i.e., known pathogenic and likely pathogenic variants in the same set of genes). This raises the question of whether there is an ethical imperative to use these chips to provide such secondary findings.

Genome-derived ancestry itself may be considered an incidental finding under some circumstances. In the polygenic setting, it is possible that an individual’s genetic ancestry will be calculated to determine which ancestry-specific PRS to use or to determine which relative risk figures to quote for the patient. If so, it is unclear whether the laboratory should report which calculated genetic ancestry was used. And if it is reported, there is a chance that this is different from how an individual might self-identify in terms of ancestry. Patient preferences should be studied about this possible unexpected finding, including how to contextualize the information.

### The role of expert mediators

Within medicine, clinical genetics is exceptional in that it leverages an allied field of genetic counseling, made up of professionals who are dedicated to helping individuals anticipate and subsequently navigate the genetic information they receive. Additionally, a patient with a known or suspected rare monogenic disorder is often seen by a specialist of that disorder. Meanwhile, some advocates of consumer-facing genetic testing for medical information maintain that individuals should be able to receive the information without an expert intermediary and should be able to get any consultation they wish to help them following this [43]. This view is controversial and would be illegal in some countries [44]. PRS thus enter a landscape where there are multiple points of view concerning the support a patient needs to understand information they are provided.

Whereas most monogenic variation of interest relates to rare disease that no one other than a specialist would be anticipated to have any experience with, PRS are being developed for common diseases that are familiar to primary care providers. Within the context of risk factors for common disease, where the inheritance of individual alleles does not severely complicate decisions, the role of both the medical geneticist and the genetic counselor could be questioned, particularly if primary care providers felt comfortable with discussing PRS with patients. To this end, further education and training for primary care providers may be necessary.

### Potential harms of genetic testing

At least three sets of potential harms are typically considered in the monogenic setting: psychosocial harms, false reassurance, and overdiagnosis and overtreatment (we consider potential genetic discrimination in a separate section below). Ethical engagement does not, however, stop at the identification of a potential harm. Rather, the process should continue to ask (a) how much the harm is realized, (b) whether it can be minimized, (c) how the suitably minimized harm compares to the benefits, and (d) how to allow an individual’s own values to determine that trade-off for themselves.

A slew of fears about the potential psychosocial harms of genetic testing have been proposed [45]. When rigorous trials to assess psychosocial harms have been conducted, many of the expected harms have not materialized. There is a near absence of negative psychological or emotional impact of genetic testing in the adult setting [46–49].

The risk of false reassurance is often put forward for those that receive false negative results; this could arise for those identified at low or average polygenic risk, though in at least some cases when evidence has been sought for false reassurance, it has not been found [50]. There are many options for how polygenic scores can be reported, including just indicating whether an individual has a score at the extreme high tail of the distribution (i.e., a binary report of identified/not identified as at high risk), or report of a continuous variable such as the PRS percentile, or a relative or absolute risk. The potential harms of false reassurance will likely depend on PRS reporting strategy, particularly while the absence of evidence-based guidelines leaves more room for interpretation by both patients and providers.

The risk of overtreatment and overdiagnosis will likely also depend on PRS reporting strategy. Patients and their providers may view any elevated-above-average polygenic risk as reason enough to initiate additional tests and even treatments, particularly if the choice is made to report a continuous variable rather than a binary variable. Other reporting options such as choice of language, infographics, and accompanying recommendations are also relevant and will also impact patient and provider reaction to PRS reports.

Data needs to be collected on whether the harms of false reassurance and overtreatment and overdiagnosis materialize. If so, steps (b)-(d) outlined above should be followed, with particular attention paid to how different reporting strategies affect both harms and benefits of clinical reporting of PRS.

### Potential concerns in using PRS in the pediatric setting

PRS can be calculated from birth. Several of the common conditions for which PRS exist, for example obesity, are ones for which lifelong health behaviors can have a sizable impact. This poses the question of the conditions under which PRS should be returned to children. Leading professional societies have adopted the position that it is not appropriate to return genetic findings to children if no childhood interventions can benefit them [51]. One basis for these positions is the possibility of psychosocial harm. Although there is considerably less data for children than adults and more data is needed, existing data do not support this fear [52]. The other basis for these positions is a child’s “right to an open future,” i.e., to have future options kept open until they can make their own decisions, an ethical argument which has recently been challenged [51]. Because many PRS exist for conditions with a broad range of ages of onset, there are likely to be lively debates about which conditions it is appropriate to return to children. A one-size-fits-all before/after age eighteen seems unlikely to fit all the conditions for which PRS exist, for example a high PRS for type 2 diabetes may not be clinically actionable for an infant, but may be for a teenager.

### Potential genetic discrimination

In the USA, fears of genetic discrimination drove major legislative efforts, culminating in the passage of the Genetic Information Non-discrimination Act (GINA) in 2008, protecting individuals against the use of genetic information in most health insurance and employment contexts [53]. GINA does not cover life, long-term care, or disability insurance, or other contexts such as lending and education. While some States have additional legislation in these areas, almost all of these permit underwriting on the basis of genetic information if that information can be convincingly linked to higher mortality or morbidity [54, 55]. While there is little documentation that genetic discrimination is widespread [56], there is evidence that individuals are put off learning their own genetic risks because of the prospect of genetic discrimination [57–59].

The issue of genetic discrimination may have renewed relevance in the PRS context, particularly if the vision is of a broad application to healthy individuals. If the data we have from research studies generalizes to the clinical setting, then we might expect many individuals to forgo the potential upsides of receiving PRS because of fear that the information will be used against them in insurance pricing or availability. Moreover, it may be that life insurers are more comfortable with using PRS to adjust premiums, as they are likely to be potent predictors on a population basis, and are formulated as exactly the sort of information that they habitually incorporate into their risk models. Life insurers would, however, have to cope with the same problems of portability and interpretation as faced by patients and physicians, so are unlikely to be early adopters of the scores.

## The intersection of public health and clinical medicine

Polygenic risk scores have been proposed both as a tool of clinical medicine and of public health. These two settings come with their own overlapping but distinct ethical frameworks. The former is focused on the individual, and patient autonomy is key. In contrast, public health is focused on the prevention or mitigation of diseases in a population. It has two main values which can compete with each other: maximizing benefit to the population and achieving equity within that population [60]. There are two main strategies for prevention, focusing on factors that affect the entire population (e.g., informational campaigns) and focusing on those identified as at highest risk [61]. There are downsides to the latter approach. First, it may not be as effective as focusing on the whole population [61]. Second, it can detract attention away from broader social determinants of health [62]. Finally, it is not straightforwardly clear what values apply in this context. We begin by examining this ambiguity, and then discuss the ethical issue that has received most attention so far, the potential for contributing to health disparities.

### What endpoints are we maximizing for, under which constraints?

Current and former members of the U.S. Preventive Services Task Force argue: “Our experience with multiple screening topics has taught us to focus on health outcomes rather than diseases or intermediate outcomes. The purpose of screening is to improve the length and/or quality of people’s lives, not just to find abnormalities” [63]. In other words, the metric by which a screening program for identifying those at higher risk of a disease should be judged is not by number of novel diagnoses but by direct effects on mortality and quality of life. The former is, however, easier to measure in clinical trials. Improvements in mortality and quality of life come not just from the information shared on the PRS report, but the ways in which the medical system facilitates appropriate follow up recommendations [19].

Even if a trial did manage to establish improvements in length or quality of life endpoints, this misses the key element of autonomy [64, 65]. After all, no change in outcome is achieved unless the patient chooses to participate. Just like the aim of prenatal screening is not a reduction in those born with disabilities but is rather an enhancement of reproductive autonomy, the aim of sharing risk factors should not be simply increased length or quality of life [66]. The language often employed is that of empowerment [67]. For example the UK’s Chief Medical Officer states in the context of genomics that health systems will need to “empower them [patients] to take greater responsibility for their health and have greater regard to their personal values and wishes” [68]. The issue of responsibility is an ethically charged one, as it is not clear to what extent we want individuals to feel responsible for their own health [69]. Answers to questions of responsibility, guilt, and blame are all of practical relevance in the tailoring of messages alongside risk information [70] and even the display of the risk information itself. For example, patient understanding and intention to change healthcare behaviors are not necessarily maximized by the same choices of how to display risk information [71]. Physicians adapt their patient interaction strategies to achieve desired results [72]; they could use PRS to motivate behavior change [73]. Key ELSI considerations are the extent and degree to which nudging (i.e., the design of the choice environment to help people make decisions that are better for themselves) is appropriate [74, 75] and, more generally, what the ethical constraints are in maximizing the proportion of people that act on risk factor findings.

### The potential for contributing to health disparities

The scientific community has drawn attention to the potential contribution of the clinical use of PRS to race-based health disparities, stemming from the current inferior performance of PRS based upon the existing largely European ancestry data in non-European ancestry populations (see the portability problem, above) [14]. This skew in data is itself the product of many factors [76]. For PRS, if current scores are beneficial, which is under active debate, and if the benefits depend on predictive power, then their use would disproportionately benefit those of European ancestry. Similar dangers have also been raised for other branches of personalized medicine [77]. Statistical methodologies have been proposed to help narrow this gap in predictive power, but they will not be able to fully close it. This poses an immediate issue of whether tests should be restricted by ancestry. This is the path taken for example by Ambry Genetics, who restrict their prostate cancer test to males of European ancestry, and their breast cancer test to females of Non-Ashkenazi Jewish, Northern European ancestry [6]. One alternative would be to make the test available to those of all ancestries, in which case the performance of the test should be reported in as many populations as possible, and the results suitably caveated. Which of these alternatives is preferable will be context dependent, but ideally, it is those negatively impacted who should have the deciding voice, for example by using focus groups [78].

The medium-term remedy identified is to gather large data sets from populations of diverse ancestries [79]. Several efforts are underway, including the NIH funded “All of Us” program, which aims to recruit more than 45% of its one million participants from racial and ethnic minorities [80]. It is unclear how we will know if these laudable efforts suitably address the identified issue. The predictive power of a biomarker is not guaranteed to be the same across different groups, even in the presence of very large samples, partly because the environment can differ systematically across these groups. Of course, the routine clinical use of PRS has the potential to exacerbate health disparities *even if* equal predictive power is obtained across different ancestry groups, for the all too familiar structural reasons that cause those disparities in the first place, including lack of access to care. A spectrum of approaches to assessing and addressing inequities will be needed.

## A spotlight on biomarkers for risk

Besides the issues raised by the use of PRS in a public health context, an additional set of concerns that may arise with the clinical use of PRS are related to long-standing issues with the use of biomarkers to identify those at high risk of disease, including the dangers of overtreatment and overdiagnosis [28]. These concerns are linked to how risk biomarkers can change what we mean by “a disease.” For example, the links between elevated blood pressure and heart disease led to the introduction of prehypertension as a disease diagnosis, a classification that applies to vast numbers of asymptomatic individuals [81]. The ways in which the distributions of biomarker values differ across populations have also led to a long-simmering debate. PRS are likely to reinvigorate both these conversations. The use of biomarkers for psychiatric conditions is not well established, and in the case of PRS, research is interwoven with sociobehavioral outcomes, posing new ethical challenges.

### The relevance of race, ethnicity, and ancestry

The argument that clinical use of PRS could contribute to health disparities discussed above stems from the current poorer performance of PRS in non-European ancestry individuals, itself largely a result of the lack of representation of these individuals in the underlying data. A separate set of concerns relates to how the concepts of race, ethnicity, and ancestry are used in the development and deployment of PRS. Outside of genetics, there is no shortage of evidence for racial and ethnic differences in association between biomarkers and disease or response to drugs, with attached calls for race-specific cutoffs and algorithms [82–84]. A prominent example is the estimated glomerular filtration rate, a surrogate for renal function, for which a “correction” for race is typically made [85]. In the risk variant context, the *APOE* ε4 risk allele for Alzheimer’s disease has been reported to have different effects by self-reported race, leading some to suggest that use of this allele as a risk factor should “adjust for race” [86]. However, the use of “race as a biological variable” is controversial, out of concerns that this construct could detract from social determinants of health, reinforce racial stereotypes, and contribute to viewing race—a social construct—as predominantly based in biology [87, 88]. Adjustments for race might be viewed as appropriate if they are tied to underlying genetic differences, suggesting the need for careful attention and research into the relationships between ancestry, race, racism, and the environment [89]. This is particularly true for PRS scores, because the portability problem means that attention to inferred genetic ancestry will be necessary to ensure that patients receive accurate and properly interpreted results. This will not be a straightforward task, as there are distinct differences in how those in population genetics, clinical medicine, and epidemiology consider the role of genetic ancestry [90]. The use of race, ancestry, and ethnicity in polygenic reporting will hence be fraught with both practical difficulties and ethical questions. Urgent interdisciplinary work to untangle some of these questions is needed.

### Biomarkers for conditions that present unique challenges

There are at least three reasons to think that PRS for psychiatric conditions may raise unique ethical concerns. The first stems from a clinical distinction: the use of any non-genetic biomarkers for risk for these conditions is very limited. The effect of sharing biomarker risk in general for psychiatric conditions is hence understudied, and the possible harms unclear. For example, there are potential harms regarding effect on personal identity that seem especially salient for psychiatric conditions [91]. Second, the phenotype definitions for psychiatric conditions that are used in GWAS studies are widely acknowledged not to capture the distinct dimensions of biological underpinnings of the different conditions [92]. PRS for psychiatric conditions have been shown to have high pleiotropy, meaning that they associate not just with the condition they are based on but for other psychiatric conditions [93]. Any clinical use of psychiatric PRS would have to consider the potential relevance to conditions other than the one explicitly tested for.

The third reason is the existence of the research agenda that looks for genetic correlations between predisposition for psychiatric conditions and various sociobehavioral traits, such as alcohol use, antisocial behavior, and intelligence (reviewed in [94]). These efforts to link the genetics of medical conditions and social outcomes expands the potential relevance of being labeled as at high risk for a psychiatric condition, an expansion which may be very unwelcome to many, and which heightens the possibility that individuals may experience stigmatization because of such a label. Much conceptual and empirical work will be needed to understand the ramifications of these links to social genomics.

### The concept of a disease

It is part of the hope of precision medicine that disease classification is refined. In this vision, the broad definitions we have of conditions, based on symptoms and test results, are replaced with delineations based on differences in a molecular taxonomy that reflects underlying biology [95]. In the monogenic setting, this involves identifying molecular subtypes of disease, a process that could prompt more accurate prognoses and potentially new treatments. In the polygenic setting, risk scores are linked to disease status via the liability-threshold model [9]. In this model, liability is a continuous estimation of summed genetic and environmental attributes related to the causes of a disease. Above a certain threshold of liability, the disease is present, or else it is absent [96]. In this model, the continuous value—liability—has no relevance except in connection with the threshold that determines whether disease is predicted to be present. Some have proposed that within psychiatry, PRS should lead us to abandon this line drawing exercise entirely, moving away from qualitative (yes/no) diagnoses—even if refined by mild/moderate/severe designations—in favor of positioning along quantitative dimensions [97]. This same logic might hold for non-psychiatric conditions. Abandoning the binary classification system could have far-reaching, long-term implications on medical practice, helping to transform the notion and the practice of healthcare from reactive to proactive, from treatment to prevention.

How disease is conceptualized also has ramifications outside medicine, for example the concept is entrenched within legislation. In the USA, the Americans with Disabilities Act covers individuals who are, or who are regarded as being, limited in a major life activity. GINA covers disease conditions that are not yet “manifested.” Those in a “pre-disease” state fall between these statutes, and hence do not benefit from the protections they offer [98]. The concept of a disease is also central to debates about the ethical permissibility of genetic modification, where the therapy/enhancement divide—which is primarily based on the concept of a disease—is often regarded as relevant by the public and by policy makers for distinguishing permissible from impermissible uses [99]. The policy implications that result from a changing concept of disease are the most speculative we have considered here, but they could also be the most far reaching.

## Conclusions

The prospect of clinical use of PRS is associated with a wide variety of ELSI concerns. Many of the issues that have been and continue to be discussed in the context of monogenic genetic results are also present in the polygenic context, albeit sometime in modified form. These include the relevance of results to family members, the approach for secondary/incidental findings, the role of expert mediators, the potential harms of testing, unique concerns for the pediatric population, and the prospect of genetic discrimination. Moreover, two additional aspects of the clinical use of PRS raise specific ELSI concerns. The first is the potential use of PRS as a tool of public health, a use case for which careful thought about what endpoints we are maximizing for is needed, and for which the impact on health disparities is central. The second is that PRS can be viewed as a biomarker for risk of common disease, an area which is already grappling with whether and how to incorporate race, ethnicity, and ancestry, issues that will be particularly acute for PRS. PRS as biomarkers for risk also raise questions about the very concept of a binary definition of disease. And particularly for PRS for psychiatric conditions, associations with sociobehavioral traits complicates their ethical use.

Given the speed with which the science has developed, and the calls for widespread clinical use of PRS, we urgently need further conceptual and empirical work to define ethically defensible best practices, to establish and track the right outcome metrics, as well as to minimize broader societal harms and unnecessary costs. Some work has already started or is planned, for example the eMERGE IV study mentioned above, which will return PRS reports to 20,000 individuals, will be informed by ELSI projects investigating many aspects of the process by which polygenic results are returned, but much more is needed.

While aspects of PRS science, particularly the relevance of population structure, seem to raise novel ethical questions, almost all the issues we identify are old ones in new wrapping. Our discussion demonstrates the continuity between polygenic risk information and other predictive health information. Ultimately, we expect PRS to help de-exceptionalize genomic information, while simultaneously drawing greater scrutiny to other predictive biological information.

## Data Availability

Not applicable

## References

[1] Khera AV, Chaffin M, Aragam KG, Haas ME, Roselli C, Choi SH (2018). Genome-wide polygenic scores for common diseases identify individuals with risk equivalent to monogenic mutations. Nat Genet.

[2] Inouye M, Abraham G, Nelson CP, Wood AM, Sweeting MJ, Dudbridge F (2018). Genomic risk prediction of coronary artery disease in 480,000 adults: implications for primary prevention. J Am Coll Cardiol.

[3] Howard DM, Adams MJ, Clarke T-K, Hafferty JD, Gibson J, Shirali M (2019). Genome-wide meta-analysis of depression identifies 102 independent variants and highlights the importance of the prefrontal brain regions. Nat Neurosci.

[4] Khera AV, Chaffin M, Wade KH, Zahid S, Brancale J, Xia R (2019). Polygenic prediction of weight and obesity trajectories from birth to adulthood. Cell.

[5] Mavaddat N, Pharoah PDP, Michailidou K, Tyrer J, Brook MN, Bolla MK, et al. Prediction of breast cancer risk based on profiling with common genetic variants. JNCI J Natl Cancer Inst. 2015;107. 10.1093/jnci/djv036. 10.1093/jnci/djv036 PMC4754625 25855707

[6] Cancer Genetic Testing | AmbryScore | Health Risk Tests | Ambry Genetics n.d. https://www.ambrygen.com/clinician/ambryscore. Accessed 12 Dec 2019

[7] riskScore. Myriad MyRisk n.d. https://myriadmyrisk.com/riskscore/. Accessed 12 Dec 2019

[8] 23andMe will tell you how your DNA affects your diabetes risk. STAT 2019. https://www.statnews.com/2019/03/10/23andme-will-tell-you-how-your-dna-affects-your-diabetes-risk-will-it-be-useful/. Accessed 18 Aug 2019

[9] Martin AR, Daly MJ, Robinson EB, Hyman SE, Neale BM (2019). Predicting polygenic risk of psychiatric disorders. Biol Psychiatry.

[10] Zhang J-P, Robinson D, Yu J, Gallego J, Fleischhacker WW, Kahn RS (2018). Schizophrenia polygenic risk score as a predictor of antipsychotic efficacy in first-episode psychosis. Am J Psychiatry.

[11] Ruderfer DM, Charney AW, Readhead B, Kidd BA, Kähler AK, Kenny PJ (2016). Polygenic overlap between schizophrenia risk and antipsychotic response: a genomic medicine approach. Lancet Psychiatry.

[12] Lewis CM, Vassos E (2020). Polygenic risk scores: from research tools to clinical instruments. Genome Med.

[13] Ward J, Graham N, Strawbridge RJ, Ferguson A, Jenkins G, Chen W, et al. Polygenic risk scores for major depressive disorder and neuroticism as predictors of antidepressant response: Meta-analysis of three treatment cohorts. Plos One. 2018;13. 10.1371/journal.pone.0203896. 10.1371/journal.pone.0203896 PMC6150505 30240446

[14] Martin AR, Kanai M, Kamatani Y, Okada Y, Neale BM, Daly MJ (2019). Clinical use of current polygenic risk scores may exacerbate health disparities. Nat Genet.

[15] Mathieson I, Scally A. What is ancestry? PLoS Genet. 2020;16(3):e1008624. 10.1371/journal.pgen.1008624. 10.1371/journal.pgen.1008624 PMC7082057 32150538

[16] Mostafavi H, Harpak A, Conley D, Pritchard JK, Przeworski M. Variable prediction accuracy of polygenic scores within an ancestry group. BioRxiv. 2019;629949. https://elifesciences.org/articles/48376. 10.7554/eLife.48376 PMC7067566 31999256

[17] Kong A, Thorleifsson G, Frigge ML, Vilhjalmsson BJ, Young AI, Thorgeirsson TE (2018). The nature of nurture: Effects of parental genotypes. Science.

[18] Young AI, Benonisdottir S, Przeworski M, Kong A (2019). Deconstructing the sources of genotype-phenotype associations in humans. Science.

[19] Raffle A, Mackie A, Gray JAM. Screening: Evidence and Practice. Second Edition, New to this Edition: Oxford. New York: Oxford University Press; 2019.

[20] Wald NJ, Old R (2019). The illusion of polygenic disease risk prediction. Genet Med.

[21] Warren M (2018). The approach to predictive medicine that is taking genomics research by storm. Nature.

[22] Khoury. Is it time to integrate polygenic risk scores into clinical practice? Let’s do the science first and follow the evidence wherever it takes us! | | Blogs | CDC 2019. https://blogs.cdc.gov/genomics/2019/06/03/is-it-time/. Accessed 17 Aug 2019.

[23] Esserman LJ (2017). The WISDOM Study: breaking the deadlock in the breast cancer screening debate. Npj Breast Cancer.

[24] RFA-HG-19-013: The Electronic Medical Records and Genomics (eMERGE): Genomic Risk Assessment and Management Network 2019. https://grants.nih.gov/grants/guide/rfa-files/rfa-hg-19-013.html. Accessed 17 Aug 2019

[25] Advancing our health: prevention in the 2020s – consultation document. GOVUK n.d. https://www.gov.uk/government/consultations/advancing-our-health-prevention-in-the-2020s/advancing-our-health-prevention-in-the-2020s-consultation-document. Accessed 6 Jan 2020

[26] Chowdhury S, Dent T, Pashayan N, Hall A, Lyratzopoulos G, Hallowell N (2013). Incorporating genomics into breast and prostate cancer screening: assessing the implications. Genet Med Off J Am Coll Med Genet.

[27] Gibson G (2019). Going to the negative: genomics for optimized medical prescription. Nat Rev Genet.

[28] Bleyer A, Welch HG. Effect of three decades of screening mammography on breast-cancer incidence. Http://DxDoiOrg/101056/NEJMoa1206809 2012. 10.1056/NEJMoa1206809. 10.1056/NEJMoa1206809 23171096

[29] Aronowitz R (2015). Risky medicine: our quest to cure fear and uncertainty.

[30] Mosley JD, Gupta DK, Tan J, Yao J, Wells QS, Shaffer CM (2020). Predictive accuracy of a polygenic risk score compared with a clinical risk score for incident coronary heart disease. JAMA.

[31] Elliott J, Bodinier B, Bond TA, Chadeau-Hyam M, Evangelou E, Moons KGM (2020). Predictive accuracy of a polygenic risk score–enhanced prediction model vs a clinical risk score for coronary artery disease. JAMA.

[32] Isgut M, Sun J, Quyyumi AA, Gibson G. Highly elevated polygenic risk scores are better predictors of myocardial infarction risk early in life than later. Genome Med n.d. 10.1186/s13073-021-00828-8 10.1186/s13073-021-00828-8 PMC7845089 33509272

[33] Kullo IJ, Hayan J, Austin Erin E, Sherry-Ann B, Kruisselbrink Teresa M, Isseh Iyad N (2016). Incorporating a genetic risk score into coronary heart disease risk estimates. Circulation.

[34] Hollands GJ, French DP, Griffin SJ, Prevost AT, Sutton S, King S (2016). The impact of communicating genetic risks of disease on risk-reducing health behaviour: systematic review with meta-analysis. BMJ.

[35] Stewart KFJ, Wesselius A, Schreurs MAC, Schols AMWJ, Zeegers MP (2018). Behavioural changes, sharing behaviour and psychological responses after receiving direct-to-consumer genetic test results: a systematic review and meta-analysis. J Community Genet.

[36] Frieser MJ, Wilson S, Vrieze S (2018). Behavioral impact of return of genetic test results for complex disease: Systematic review and meta-analysis. Health Psychol Off J Div Health Psychol Am Psychol Assoc.

[37] Widen E, Aro J, Pollanen P, Hotakainen K, Partanen J, Ripatti S (2018). Receiving personal genome-based disease risk information motivates individuals to take action to prevent cardiovascular disease (CVD). (#270).

[38] Holm IA, McGuire A, Pereira S, Rehm H, Green RC, Beggs AH (2019). Returning a genomic result for an adult-onset condition to the parents of a newborn: insights from the BabySeq Project. Pediatrics.

[39] Taub S, Morin K, Spillman MA, Sade RM, Riddick FA, Council on Ethical and Judicial Affairs of the American Medical Association. Managing familial risk in genetic testing. Genet Test 2004;8:356–359. 10.1089/gte.2004.8.356. 10.1089/gte.2004.8.356 15727262

[40] Karavani E, Zuk O, Zeevi D, Barzilai N, Stefanis NC, Hatzimanolis A (2019). Screening human embryos for polygenic traits has limited utility. Cell.

[41] Green RC, Berg JS, Grody WW, Kalia SS, Korf BR, Martin CL (2013). ACMG recommendations for reporting of incidental findings in clinical exome and genome sequencing. Genet Med Off J Am Coll Med Genet.

[42] Burke W, Matheny Antommaria AH, Bennett R, Botkin J, Clayton EW, Henderson GE, et al. Recommendations for returning genomic incidental findings? We need to talk! Genet Med Off J Am Coll Med Genet. 2013;15. 10.1038/gim.2013.113. 10.1038/gim.2013.113 PMC3832423 23907645

[43] Wojcicki, Anne. Consumers don’t need experts to interpret 23andMe genetic risk reports. STAT 2018. https://www.statnews.com/2018/04/09/consumers-23andme-genetic-risk-reports/. Accessed 12 Jan 2020.

[44] Thorogood A, Dalpé G, Knoppers BM (2019). Return of individual genomic research results: are laws and policies keeping step?. Eur J Hum Genet.

[45] Clayton EW (2003). Ethical, legal, and social implications of genomic medicine. N Engl J Med.

[46] Robinson JO, Wynn J, Biesecker B, Biesecker LG, Bernhardt B, Brothers KB (2019). Psychological outcomes related to exome and genome sequencing result disclosure: a meta-analysis of seven Clinical Sequencing Exploratory Research (CSER) Consortium studies. Genet Med.

[47] Oliveri S, Ferrari F, Manfrinati A, Pravettoni G. A systematic review of the psychological implications of genetic testing: a comparative analysis among cardiovascular, neurodegenerative and cancer diseases. Front Genet. 2018;9. 10.3389/fgene.2018.00624. 10.3389/fgene.2018.00624 PMC6295518 30619456

[48] Parens, Erik, Appelbaum, Paul. Disclosing genetic information: not as worrisome as once feared. STAT 2019. https://www.statnews.com/2019/07/30/genetic-information-disclosure/.

[49] Roberts JS (2019). Assessing the psychological impact of genetic susceptibility testing. Hastings Cent Rep.

[50] Olfson E, Hartz S, Carere DA, Green RC, Roberts JS, Bierut LJ (2016). Implications of personal genomic testing for health behaviors: the case of smoking. Nicotine Tob Res Off J Soc Res Nicotine Tob.

[51] Garrett JR, Lantos JD, Biesecker LG, Childerhose JE, Chung WK, Holm IA (2019). Rethinking the “open future” argument against predictive genetic testing of children. Genet Med.

[52] Wakefield CE, Hanlon LV, Tucker KM, Patenaude AF, Signorelli C, McLoone JK (2016). The psychological impact of genetic information on children: a systematic review. Genet Med Off J Am Coll Med Genet.

[53] Genetic Information Nondiscrimination Act of 2008 n.d. https://www.eeoc.gov/laws/statutes/gina.cfm. Accessed 13 Jan 2020

[54] Wolf LE, Fuse Brown E, Kerr R, Razick G, Tanner G, Duvall B (1991). The web of legal protections for participants in genomic research. Health Matrix Clevel Ohio.

[55] Prince A (2018). Insurance risk classification in an era of genomics: is a rational discrimination policy rational?. Neb Law Rev.

[56] Green RC, Lautenbach D, McGuire AL (2015). GINA, genetic discrimination, and genomic medicine. N Engl J Med.

[57] Robinson JO, Carroll TM, Feuerman LZ, Perry DL, Hoffman-Andrews L, Walsh RC (2016). Participants and study decliners’ perspectives about the risks of participating in a clinical trial of whole genome sequencing. J Empir Res Hum Res Ethics JERHRE.

[58] Genetti CA, Schwartz TS, Robinson JO, VanNoy GE, Petersen D, Pereira S, et al. Parental interest in genomic sequencing of newborns: enrollment experience from the BabySeq Project. Genet Med. 2018;1. 10.1038/s41436-018-0105-6. 10.1038/s41436-018-0105-6 PMC6420384 30209271

[59] Amendola LM, Robinson JO, Hart R, Biswas S, Lee K, Bernhardt BA (2018). Why patients decline genomic sequencing studies: experiences from the CSER Consortium. J Genet Couns.

[60] Barrett DH, Ortmann LH, Dawson A, Saenz C, Reis A, Bolan G, editors. Public health ethics: cases spanning the globe. Springer International Publishing; 2016. doi: 10.1007/978-3-319-23847-0. 28590613

[61] Rose G (2001). Sick individuals and sick populations. Int J Epidemiol.

[62] Chowkwanyun M, Bayer R, Galea S (2018). “Precision” public health — between novelty and hype. N Engl J Med.

[63] Harris R, Sawaya GF, Moyer VA, Calonge N (2011). Reconsidering the criteria for evaluating proposed screening programs: reflections from 4 current and former members of the U.S. Preventive Services Task Force. Epidemiol Rev.

[64] Rogowski W, Payne K, Schnell-Inderst P, Manca A, Rochau U, Jahn B (2015). Concepts of “personalization” in personalized medicine: implications for economic evaluation. PharmacoEconomics.

[65] Pravettoni G, Gorini A (2011). A P5 cancer medicine approach: why personalized medicine cannot ignore psychology. J Eval Clin Pract.

[66] Godard B, ten Kate L, Evers-Kiebooms G, Aymé S (2003). Population genetic screening programmes: principles, techniques, practices, and policies. Eur J Hum Genet.

[67] Junegst E, Flatt M, Setterson R (2012). Personalized genomic medicine and the rhetoric of empowerment. Hastings Cent Rep.

[68] Chief Medical Officer annual report 2016: generation genome. GOVUK 2016. https://www.gov.uk/government/publications/chief-medical-officer-annual-report-2016-generation-genome. Accessed 16 Sept 2019

[69] Wikler D (2002). Personal and social responsibility for health. Ethics Int Aff N Y.

[70] Guttman N, Salmon CT (2004). Guilt, fear, stigma and knowledge gaps: ethical issues in public health communication interventions. Bioethics.

[71] Witteman HO, Fuhrel-Forbis A, Wijeysundera HC, Exe N, Dickson M, Holtzman L (2014). Animated randomness, avatars, movement, and personalization in risk graphics. J Med Internet Res.

[72] Bonner C, Jansen J, McKinn S, Irwig L, Doust J, Glasziou P (2014). Communicating cardiovascular disease risk: an interview study of General Practitioners’ use of absolute risk within tailored communication strategies. BMC Fam Pract.

[73] Vassy JL, Christensen KD, Slashinski MJ, Lautenbach DM, Raghavan S, Robinson JO (2015). ‘Someday it will be the norm’: physician perspectives on the utility of genome sequencing for patient care in the MedSeqProject. Pers Med.

[74] Thaler RH, Sunstein CR (2009). Nudge: improving decisions about health, wealth, and happiness. Revised&Expanded edition.

[75] Noggle R. The Ethics of Manipulation. In: Zalta EN, editor. Stanf. Encycl. Philos. Summer 2018, Metaphysics Research Lab, Stanford University; 2018. https://plato.stanford.edu/entries/ethics-manipulation/.

[76] Hussain-Gambles M, Atkin K, Leese B (2004). Why ethnic minority groups are under-represented in clinical trials: a review of the literature. Health Soc Care Community.

[77] Duconge J, Ruaño G (2018). Preventing the exacerbation of health disparities by iatrogenic pharmacogenomic applications: lessons from warfarin. Pharmacogenomics.

[78] Christensen KD, Roberts JS, Royal CDM, Fasaye G-A, Obisesan T, Cupples LA (2008). Incorporating ethnicity into genetic risk assessment for Alzheimer disease: the REVEAL study experience. Genet Med Off J Am Coll Med Genet.

[79] Genetics for all. Nat Genet 2019;51:579–579. 10.1038/s41588-019-0394-y. 10.1038/s41588-019-0394-y 30926967

[80] The “All of Us” Research Program. N Engl J Med 2019;381:668–676. doi: 10.1056/NEJMsr1809937. 10.1056/NEJMsr1809937 PMC8291101 31412182

[81] Nary FC, Santos RD, Laurinavicius AG, Conceição RD de O, de Carvalho JAM. Relevance of prehypertension as a diagnostic category in asymptomatic adults. Einstein 2013;11:303–309. 10.1590/S1679-45082013000300008. 10.1590/S1679-45082013000300008 PMC4878588 24136756

[82] Gijsberts CM, Seneviratna A, den Ruijter HM, Asselbergs FW, Agostoni P, Bank IEM (2016). The ethnicity-specific association of biomarkers with the angiographic severity of coronary artery disease. Neth Heart J.

[83] Veeranna V, Zalawadiya SK, Niraj A, Kumar A, Ference B, Afonso L (2013). Association of novel biomarkers with future cardiovascular events is influenced by ethnicity: results from a multi-ethnic cohort. Int J Cardiol.

[84] Rappoport N, Paik H, Oskotsky B, Tor R, Ziv E, Zaitlen N (2018). Comparing ethnicity-specific reference intervals for clinical laboratory tests from EHR data. J Appl Lab Med.

[85] Omuse G, Maina D, Mwangi J, Wambua C, Kanyua A, Kagotho E (2017). Comparison of equations for estimating glomerular filtration rate in screening for chronic kidney disease in asymptomatic black Africans: a cross sectional study. BMC Nephrol.

[86] Morris JC, Schindler SE, McCue LM, Moulder KL, Benzinger TLS, Cruchaga C (2019). Assessment of racial disparities in biomarkers for Alzheimer disease. JAMA Neurol.

[87] Sankar P, Cho MK, Condit CM, Hunt LM, Koenig B, Marshall P (2004). Genetic research and health disparities. JAMA J Am Med Assoc.

[88] Roberts DE. Law, race, and biotechnology: toward a biopolitical and transdisciplinary paradigm. Annu Rev Law Soc Sci 2013;9:149–166. doi: 10.1146/annurev-lawsocsci-102612-134009.

[89] Vyas DA, Eisenstein LG, Jones DS (2020). Hidden in plain sight — reconsidering the use of race correction in clinical algorithms. N Engl J Med.

[90] Yu J-H, Taylor JS, Edwards KL, Fullerton SM (2012). What are our AIMs? Interdisciplinary perspectives on the use of ancestry estimation in disease research. AJOB Prim Res.

[91] Singh I, Rose N (2009). Biomarkers in psychiatry. Nature.

[92] Dissecting the phenotype in genome-wide association studies of psychiatric illness. Br J Psychiatry J Ment Sci 2009;195:97–99. doi: 10.1192/bjp.bp.108.063156. 10.1192/bjp.bp.108.063156 PMC4739810 19648536

[93] Zheutlin AB, Dennis J, Karlsson Linnér R, Moscati A, Restrepo N, Straub P (2019). Penetrance and pleiotropy of polygenic risk scores for schizophrenia in 106,160 patients across four health care systems. Am J Psychiatry.

[94] Palk AC, Dalvie S, de Vries J, Martin AR, Stein DJ (2019). Potential use of clinical polygenic risk scores in psychiatry – ethical implications and communicating high polygenic risk. Philos Ethics Humanit Med.

[95] Khoury MJ, Gwinn ML, Glasgow RE, Kramer BS (2012). A population approach to precision medicine. Am J Prev Med.

[96] Falconer DS (1965). The inheritance of liability to certain diseases, estimated from the incidence among relatives. Ann Hum Genet.

[97] Plomin R. Blueprint: how DNA makes us who we are. MIT Press 2018. https://mitpress.mit.edu/books/blueprint. Accessed 28 Oct 2018

[98] Suter S (2015). The problems of liminal states, line drawing, and false dichotomies. J Law Biosci.

[99] McGee A (2020). Using the therapy and enhancement distinction in law and policy. Bioethics.

